# Management of trichotillomania

**DOI:** 10.4103/0019-5545.43063

**Published:** 2005

**Authors:** Harprit Kaur, B.S. Chavan, Lok Raj

**Affiliations:** * Clinical Psychologist, Government Medical College Hospital, Sector 32, Chandigarh 160047; ** Professor, Government Medical College Hospital, Sector 32, Chandigarh 160047; *** Senior Lecturer, Government Medical College Hospital, Sector 32, Chandigarh 160047

**Keywords:** Trichotillomania, obsessive–compulsive disorder, behaviour therapy, impulsivity and compulsivity

## Abstract

Hallopeau, a French dermatologist, coined the term trichotillomania (TM) to describe alopecia (baldness) caused by self-traction of the hair, but the term now encompasses the entire syndrome of pathological hair-pulling. It is a disorder of impulse control. The authors present three (adult and child) cases of TM managed successfully using a combination of pharmacotherapy and a package of behaviour therapy. Some psychopathological aspects of the disorder are also discussed.

## INTRODUCTION

Trichotillomania (TM) is defined by DSM-IV or ICD-l 0 as a recurrent failure to resist the impulse to pull hair and with rising tension followed by relief or gratification after pulling out hair. Alternative formulations view TM as an internalizing disorder or an obsessive–compulsive spectrum disorder. The hair may be pulled from a variety of sites including the scalp, eyelashes, eyebrows, pubic region, face or any other part of the body, but the scalp is the most commonly involved.^1^ Most patients pull hair from more than one site.^2^ The onset of TM is generally in childhood or adolescence. The disorder usually runs a chronic course.^2^

TM is frequently associated with obsessive–compulsive disorder (OCD), anankastic personality disorder, borderline personality disorder and depressive disorders.^3^ It may also be associated with mental subnormality, generalized dementia, general paralysis of the insane (GPI) and postencephalitic syndromes.^4^ OCD has pharmacological similarities with TM. There is also an overlap in co-morbidity, family history of the psychiatric disorder and treatment response.^5^ The two disorders, i.e. TM and OCD, show a compulsivity versus impulsivity dimension, with TM being significantly higher in impulsiveness.^6^ Current treatment options are similar to those for OCD including a variety of medications, particularly selective serotonin reuptake inhibitors (SSRIs) and beha-vioural techniques. Studies have shown that in TM, pharmaco-therapy has not shown consistent results in terms of long-term gains.^7^ Behaviour therapy has been found to be more effective. The techniques used in behaviour therapy are habit reversal, self-monitoring, response prevention, progressive muscle relaxation, and use of pleasant imagery.^8^ Mixed results have been reported using insight-oriented psychotherapy. Kumar *et al.*^4^ report failure in a case while Aggarwal *et al.*^9^ report complete recovery which was maintained till 1-year follow-up.

Three cases of TM which were successfully treated using a combination of pharmacological and behavioural interventions similar to those used in the management of OCD are reported. Although the duration of therapy was brief, the results have been encouraging.

## CASE I

The patient is a 12-year-old male, a student of standard V, with an urban background and belonging to a family of low socioeconomic status. He presented with a 6-year history of pulling the hair from different sites of his body. He was unable to resist the urge to pull out hair, especially from the scalp and eyebrows. Hair-pulling was restricted to times when he was alone and not engaged in any activity. He was teased by his peers as there were hairless patches on the scalp. The mother reported that his food intake had decreased and he had become withdrawn.

The patient was started on clomipramine. There was no improvement even after a 6-week trial of clomipramine. Subsequently, behaviour therapy was started along with clomipramine. Behaviour therapy included modified Jacobson's Progressive Muscular Relaxation (modified for children), deep breathing exercises, distraction techniques, response prevention, thought-stopping and diary maintenance.

Relaxation and deep breathing exercises were carried out twice a day (morning and evening). The patient maintained a record of the frequency, site and number of hair pulled per day. He also recorded the frequency of the impulse to pull hair and those that he was successful in stopping (response prevention). When the patient had the urge, he tried to distract himself by mental calculations (e.g. serial subtractions or additions), or by engaging in an activity, e.g. clenching his fists tightly and then opening them one finger at a time, counting and looking at them till the urge of pulling the hair had completely disappeared.

The patient was positively reinforced through appreciation and acknowledgement by the parents and therapist. The credit for success was given to the patient's efforts and self-control.

The patient reported significant improvement within 2 weeks of treatment. He reported 70% improvement while the family perceived 80% improvement. Within 2 weeks, the frequency of hair-pulling reduced to once in a week, against 20–25 times at the beginning of treatment. At the last follow-up after 9 months of treatment, the patient said that he had no urge to pull hair. This was also corroborated by the family.

## CASE II

A 31-year-old married woman from a middle socioeconomic status and urban background, reported a history of plucking hair from the scalp for the past 12 years. The problem started just after she joined college when she was under stress because of studies and had persisted ever since. Hair-plucking was persistent throughout the day, especially in the afternoons when she was relatively free from household tasks. The patient also reported anxiety symptoms over trivial matters. She was highly distressed about the hairless patches on her scalp. She sought treatment because her daughter had developed the same symptoms and she blamed herself for her daughter's problem. The patient was started on fluoxetine 20 mg per day which was gradually increased to 40 mg/day. After 4 weeks of treatment, the patient reported only mild improvement. Behavioural intervention was started after 6 weeks which included Jacobson Progressive Muscular Relaxation, distraction, response prevention and thought-stopping. These were similar to those used for Case I.

The patient was similarly reinforced through appreciation and acknowledgement by her husband and therapist. Success was strongly attributed to the patient's efforts and self-control.

Before she started treatment, the patient was plucking hair 10–25 times/day. The patient and her husband reported signi-ficant improvement (about 70%) within two weeks of treatment. The patient became symptom-free within 6 weeks and has maintained this during the past 8 months of follow up. The symptoms reappeared briefly after 6 months when she was under stress, as her husband was out of station and the children became ill. However, she was able to control her symptoms within a week.

## CASE III

The patient was a 3-year-old female, the daughter of Case II. For the past 1 year, the patient had been pulling the hair from the frontotemporal area on the right side of the scalp. She had developed large, bald patches. The patient was observed to be clinging to the mother and showed signs of anxiety. The symptoms had appeared after the patient witnessed a fire accident.

The child was treated with a combination of play therapy and behavioural counselling of the parents who became the co-therapists. Specifically, through the medium of play, the patient was helped to overcome her anxiety and gain self-confidence. The parents utilized adequate disciplining and the principle of differential reinforcement, i.e. punishment and negative reinforcement for the unwanted behaviour of pulling the hair, and positive reinforcement when she stopped it. In the process, the maladaptive learnt behaviour of anxiety reduction through pulling of hair was made extinct. The improvement in the mother who may have been acting as the role model further facilitated the child's improvement and progress. The parents were asked to give the negative rein-forcement immediately, and an explanation for the same. This was largely by verbal reproach. Parents were taught how to distract the child at such times. The energies were chanellized in play activities and drawing; and these were used to build her confidence.

The symptoms gradually reduced, and the child became symptom-free within 1 month. She has maintained her symptom-free status for the past 8 months.

## DISCUSSION

In all the cases, a significant improvement was found within 3–4 weeks of intervention, which was maintained for over 9 months. The treatment package was a combination of pharmacotherapy and behaviour therapy.

There are few reported cases in the Indian context. Aggarwal *et al.*^9^ used insight-oriented psychotherapy with drug management but found minimal improvement. Rangaswami, however, reported success in a case using cognitive–behavioural therapy.^8^

Peterson *et al.*^10^ in their review reported that TM can be effectively treated with behavioural or pharmacological approaches. However, Streichemswin and Thornby^11^ found no significant difference between fluoxetine and placebo treatments in their long-term, double-blind, cross-over trial of patients with TM. Also, Cohen *et al.*^1^ in their survey study found only minimal improvement in all modalities of treatment and no significant difference in response to psychotherapy, behaviour therapy, clomipramine or fluoxetine.

The issue is thus far from being resolved. In the cases discussed above, the results were very encouraging, and the patients and their families expressed high satisfaction with the treatment.

The methods used with these cases are techniques that have been confirmed to be efficacious in treating OCD. McElroy *et al.*^5^ in their review made a case for TM to be considered an OCD spectrum disorder. Neurobiological investigations have paralleled aetiological studies of OCD and have demonstrated both similarities and differences between these two disorders.^2^ Further studies on larger groups, using behaviour therapy show a closeness between the two. Thus, controlled studies for the same are highly recommended.

Cohen *et al.*^1^ reported that 58% of cases of TM never seek treatment. In the discussed cases too, the problem was long-standing (6–12 years), and treatment was sought for dermatological complications rather than the problem *per se.* It strengthens the case for the liaison of psychiatry with other specialties, as well as creating awareness about the disorder.

Schlosser *et al.*^12^ reported that about 5% of first-degree relatives of patients with TM are hair pullers. It would be worthwhile exploring whether it is an isolated symptom which is a learnt behaviour, or it fulfils the criteria for a diagnosis of TM. Case III might fall into this category.
